# *dxtbx*: the diffraction experiment toolbox

**DOI:** 10.1107/S1600576714011996

**Published:** 2014-07-19

**Authors:** James M. Parkhurst, Aaron S. Brewster, Luis Fuentes-Montero, David G. Waterman, Johan Hattne, Alun W. Ashton, Nathaniel Echols, Gwyndaf Evans, Nicholas K. Sauter, Graeme Winter

**Affiliations:** a Diamond Light Source Ltd, Harwell Science and Innovation Campus, Didcot OX11 0DE, UK; b Lawrence Berkeley National Laboratory, 1 Cyclotron Road, Berkeley, CA 94720, USA; c STFC Rutherford Appleton Laboratory, Didcot OX11 0FA, UK; d CCP4, Research Complex at Harwell, Rutherford Appleton Laboratory, Didcot OX11 0FA, UK

**Keywords:** single-crystal X-ray diffraction, data processing, computer programs

## Abstract

Data formats for recording X-ray diffraction data continue to evolve rapidly to accommodate new detector technologies developed in response to more intense light sources. Processing the data from single-crystal X-ray diffraction experiments therefore requires the ability to read, and correctly interpret, image data and metadata from a variety of instruments employing different experimental representations. Tools that have previously been developed to address this problem have been limited either by a lack of extensibility or by inconsistent treatment of image metadata. The *dxtbx* software package provides a consistent interface to both image data and experimental models, while supporting a completely generic user-extensible approach to reading the data files. The library is written in a mixture of C++ and Python and is distributed as part of the *cctbx* under an open-source licence at http://cctbx.sourceforge.net.

## Abstract 2

A Python/C++ library for reading image data and experimental geometry for X-ray diffraction experiments from arbitrary data sources is presented.

## 1. Introduction   

Effective processing of X-ray diffraction data from single-crystal diffraction experiments relies on an accurate model of the experimental geometry, which in turn depends on the ability to read, with no loss of information, the wide variety of data formats used for X-ray diffraction experiments. While many experiments for macromolecular crystallography employ a simple geometry (rotation axis perpendicular to the direct beam, coincident with one detector axis and in which the ‘beam centre’ is somewhere near the middle of the detector), the general diffraction experiment may employ a much more complex geometry, allowing for arbitrary positioning of a complex detector and the sample rotation axis. For example, the experiment may employ multi-axis goniometry or have a complex detector composed of multiple noncoplanar sensor panels (such as the Pilatus 12M-DLS used on Diamond beamline I23). Reliable reproduction of this geometry from a range of different descriptions requires both a standardized representation and the ability to import the experimental geometry from a variety of instruments. This is complicated by the possibility of storing the information in different ways, *e.g.* expressing the beam centre in pixels or millimetres, or with different coordinate system conventions. While universal adoption of standards such as imgCIF (Bernstein & Hammersley, 2005 ▶) for the recording of X-ray diffraction data could resolve these challenges, historical precedent indicates that this is unlikely.

The task of developing a tool to uniformly read diffraction image headers and data has been addressed more than once. The *CCP4* DiffractionImage library (Remacle & Winter, 2007 ▶) was developed to support the *DNA* (Leslie *et al.*, 2002 ▶) and *xia2* (Winter, 2010 ▶) projects, as it was realized early on that reliable access to a range of image headers was vital. This was, however, limited by a lack of extensibility and by assumptions made early in the design that the experimental geometry would correspond to the simple layout described above. The Computational Crystallography Toolbox (*cctbx*) (Grosse-Kunst­leve *et al.*, 2002 ▶) includes a package, *iotbx.detectors*, providing data access for the indexing program *LABELIT* (Sauter *et al.*, 2004 ▶) and the X-ray free-electron laser (XFEL) data analysis program *cctbx.xfel* (Sauter *et al.*, 2013 ▶), yet it suffers similar limitations. More recent efforts, such as *FabIO* (Knudsen *et al.*, 2013 ▶), help to allow general access to the data but have less emphasis on the metadata so critical for crystallographic data and their analysis.

Here we present the diffraction experiment toolbox (*dxtbx*), a software toolkit within the *cctbx* for writing new diffraction data visualization and analysis applications, which has the aim of allowing a completely general and user-extensible approach to the reading and interpretation of diffraction image data and metadata. The *dxtbx* follows the principle that the interpretation and analysis of X-ray diffraction data should be distinct and separable. This design allows the *dxtbx* to be generally applicable to the reading of X-ray diffraction data and metadata and will help to liberate developers of data processing software from the often tedious task of supporting multiple file formats and data representations within their applications. The *dxtbx* is written in a mixture of C++ and Python and is distributed as part of the *cctbx* under an open-source licence at http://cctbx.sourceforge.net.

## 2. Method of operation   

Early in the development of the *dxtbx*, it was recognized that, in order to be generally applicable, a library for reading diffraction image headers and data must satisfy the following requirements.

(1) It must have the ability to read image data and metadata from a wide variety of detectors employing different file formats and experimental conventions.

(2) The image data and metadata must be accessible *via* a single unified interface.

(3) The library must be user-extensible without requiring modification of the library source code.

(4) Finally, the models used to represent the experiment must be able to accurately capture the detector physics (*e.g.* distortion corrections) while being sufficiently general to encompass a wide variety of diffraction measurement setups.

To achieve these aims, the *dxtbx* implements an extensible plugin framework, where beamline scientists and developers can add their own modules to handle input from different file formats with different file representations. At the cost of writing a small amount of Python code (see examples in Appendices *A* [App appa] and *B* [App appb]), the user may extend the library to support any bespoke file format and transform the metadata therein to correspond to the standard representation that is used within the *dxtbx* experimental models, which has been adopted from the imgCIF standard. A simple high-level interface that enables access to data from an entire sequence of images is also provided.

Following the methodology of the *cctbx*, the library is a hybrid system written in C++ and Python (Abrahams & Grosse-Kunstleve, 2003 ▶). Python lends itself well to rapid development, with an emphasis on clean portable code, and has an extensive standard library. Various language features facilitate the easy implementation of generic code with interchangeable components. There is, however, a performance overhead with the use of Python, owing to the interpreted nature of the language, so the experimental models were implemented in C++ to allow them to be used directly within compiled code, thereby avoiding this overhead. This means that, while only Python applications can take full advantage of the power of the *dxtbx*, compiled C++ applications and libraries can still employ the generic experimental models. The *boost.python* language binding framework is used to export the C++ interface for use in Python.

**Figure 1:**
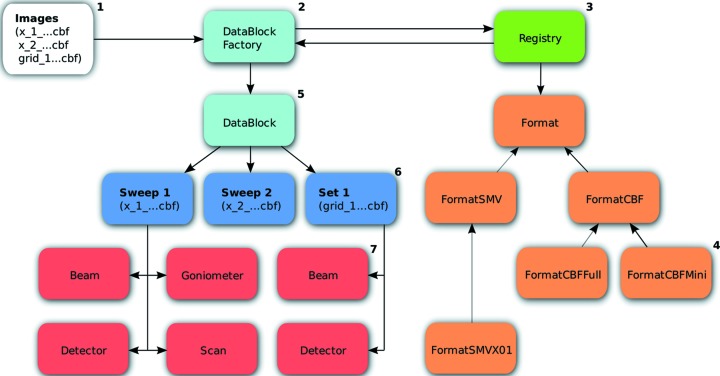
The *dxtbx* data model for a complex set of input images. The image files (1) are fed into the data block factory (2). The data block factory then uses the format registry (3) to interrogate each image to find the 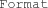 class (4) that best understands it. Note that the *dxtbx* supports more 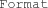 classes than are shown in the figure. If all the images use the same 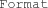 class, then a single data block (5) is returned; otherwise, multiple data blocks are created. The data block analyses the image metadata to group the images together on the basis of the set of experimental models that are shared between them. These groups can be accessed from the datablock as either sweeps or sets (6). A sweep must contain a beam, detector, goniometer and scan (7), and is thus appropriate for rotation photography; an imageset must have a beam and detector model for each image, as for a set of still shots.

The diffraction experiment toolbox consists of four distinct components: the experimental models, the high-level DataBlock and ImageSet interfaces, and the Format plugin system (§2.4). These components are described in more detail below and their interaction is illustrated in Fig. 1 ▶.

### 2.1. Experimental models   

The *dxtbx* uses the concept of experimental models to encapsulate certain aspects of the experimental description that are separable with respect to one another. The experimental models are encoded in four container classes: the beam, goniometer, detector and scan. These contain information about the source wavelength and direction, the axis about which the crystal is rotated (for rotation data), the instrument performing the measurements, and the relationship between the image frames and any rotation, respectively. In the context of single-crystal X-ray diffraction, the models are completely general with respect to experimental technique and beamline hardware. This is achieved by employing a fully vectorial description that expresses only the abstract geometry of the experiment and not other properties. No assumptions are made about the geometry besides the intersection of the beam with the crystal and the rotation axis. In particular, the rotation axis is not assumed to be orthogonal to the direction of the beam in the representation of a rotation method scan. As the models consist of vector descriptions, in principle, their components may be expressed in any chosen coordinate system; however, within the *dxtbx*, the geometry is expressed using the standard imgCIF conventions (Bernstein & Hammersley, 2005 ▶). We take many ideas from the proposals described in the EEC Cooperative Programming Workshop on Position-Sensitive Detector Software (Bricogne, 1987 ▶). In particular, we adopt the scheme for ‘virtualization’ discussed therein, which involves forming an abstract and general definition for every component of the diffraction experiment. The *dxtbx* forms the basis of the ‘instrument definition language’ outlined at that workshop, by which actual beamline hardware is mapped to its abstract model representation for any particular experiment.

**Figure 2:**
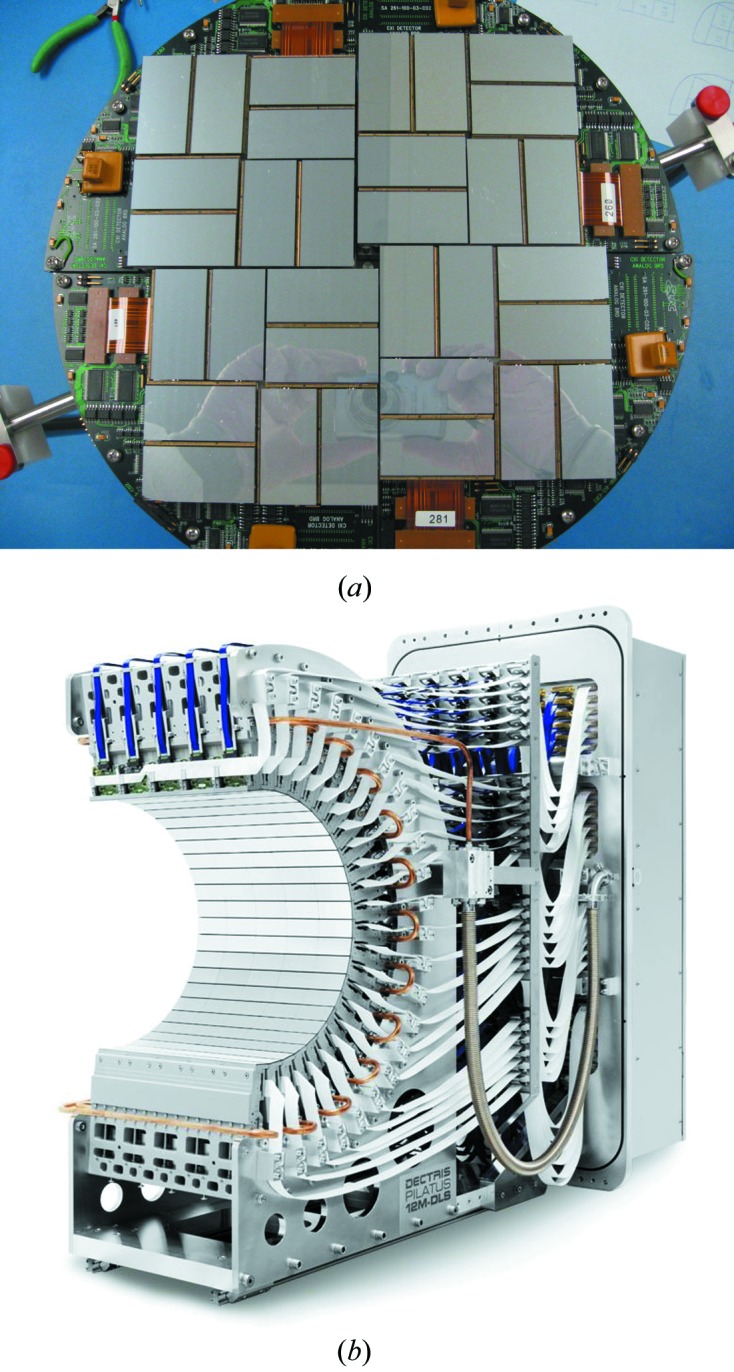
The CSPAD detector at the LCLS CXI beamline (*a*) (courtesy of Philip Hart) and the Pilatus 12M-DLS at Diamond Beamline I23 (*b*) (courtesy of DECTRIS Ltd).

Of the core experimental models, the detector model is necessarily the most complex and requires further explanation. The basic unit of our abstraction is a panel, which represents a rectangular detector plane,^1^ oriented in laboratory space. Even the simple case where the detector is a container of one or more such panels, none of which need to be coplanar, can accurately capture the half-barrel-shaped Pilatus 12M-DLS constructed for Diamond beamline I23 (Fig. 2 ▶). For more exotic detectors, the *dxtbx* supports a general hierarchical model, allowing panels to be organized into logically related groups and subgroups. This is necessary for the CSPAD (Hart *et al.*, 2012 ▶), used on the LCLS CXI beamline (Fig. 2 ▶), where subsets of panels may move with respect to each other.

In determining a position on the detector, the *dxtbx* uses the concept of a virtual detector plane. A position on the virtual plane is given by the panel identifier and a coordinate in the two-dimensional Cartesian frame attached to that panel. This point corresponds to the position at which photons impinge on the surface of the detector and is independent of the actual detector hardware in use. Behind the virtual plane interface, the hardware-specific mapping between panel position and pixel location is encapsulated within a millimetre-to-pixel function (and its inverse), which must be supplied by code specific to the actual detector hardware. This will, for example, take into account detectors with thick sensors, where the interaction point within the sensor may alter the pixel position of the measurement. In the *dxtbx*, this is realized by pairing the abstract detector model with a ‘strategy’ class (Gamma *et al.*, 1994 ▶), which allows the behaviour of the detector model to be modified without changing the model itself. This class is the natural place for all hardware-specific distortions from the simple mapping, including parallax and geometrical distortion effects, for example caused by an optical fibre taper.

**Figure 3:**
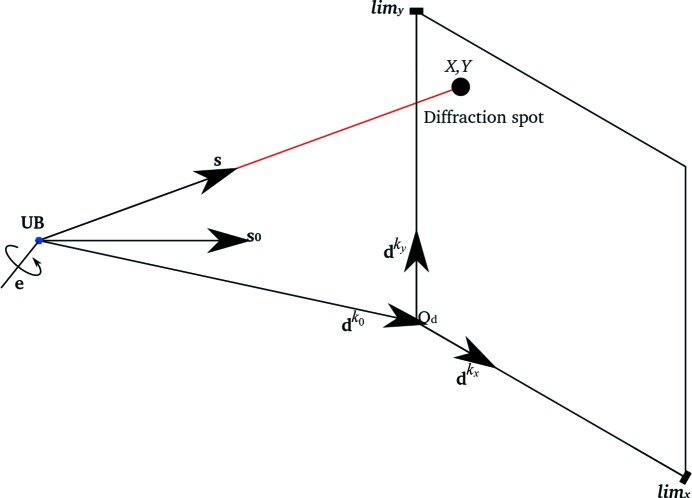
The description of diffraction geometry for the rotation method using *dxtbx* models. A monochromatic X-ray beam is represented by the wavevector 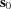, which intersects a sample rotation axis, given by the unit vector 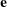, at the origin of the reciprocal laboratory coordinate system. An abstract detector plane *k* is described in the real space laboratory coordinate system with an origin vector 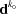 and a pair of orthogonal basis vectors 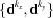. The detector model provides a pair of limits, 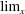 and 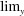, forming a bounded rectangular panel within the plane. A crystal model complements the *dxtbx* geometry models, with its setting expressed in a ϕ-axis frame (aligned to the reciprocal laboratory frame at a rotation angle of ϕ = 0°) by the setting matrix 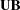, following the Protein Data Bank (http://www.pdb.org/pdb/home/home.do) convention. Diffraction is represented by the wavevector 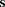, which may be extended to the point 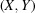 at which it meets the detector panel, in the panel’s coordinate frame.

The geometry of a single panel *k* is conveniently expressed by the matrix, 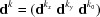. For panel *k*, the columns of the matrix are the panel basis vectors 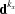 and 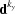, augmented by the translation vector 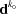, locating the origin of the panel frame in laboratory space (Fig. 3 ▶). The use of matrix 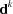 conveniently simplifies the equation for reflection prediction to a projection along a scattered direction to the detector plane, completely avoiding trigonometric functions in favour of matrix operations (Thomas, 1992 ▶). In general, all algorithms that use the *dxtbx* models do so *via* the vectorial representations summarized in Fig. 3 ▶. This ensures that the choice of coordinate frame is independent of the working of those algorithms, with the caveat that the origin of the laboratory frame is located at the intersection of the primary beam and the sample.

### 2.2. High-level DataBlock and ImageSet interfaces   

Access to the image data is provided through the high-level DataBlock interface. The data block inspects the image file metadata header information to determine the relationship between consecutive images in the list it has been provided. This enables images to be accessed as blocks of related images, such as those which share a particular set of experimental models. Blocks of images are organized according to this scheme as image sweeps and image sets. The 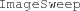 class represents a series of images that have a well defined geometric relationship between adjacent pixels in three dimensions, *e.g.* a series of images taken using the rotation method. The 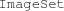 class is used where this relationship does not exist, *e.g.* for still image data resulting from serial femtosecond crystallography, but the images are nonetheless part of a single data collection. The 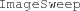 class is derived from the 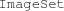 class. Both classes provide convenient access to image data through a Python list-style interface, where images in the set can be iterated over and subsets can be selected and used. The 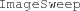 class provides additional methods to operate over a range of geometrically related images. The 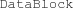 class can then contain multiple instances of the 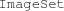 and 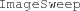 classes.

Internally, the 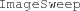 and 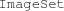 classes retain a reference to either a single- or a multi-file reader class that handles the reading of a sequence of images from a single file, such as an HDF5 file (The HDF Group, 2010 ▶), or multiple files, such as a collection of image files. Both reader classes implement a single interface, allowing the image sweep and image set to interact with different data storage representations in a generic way. Support for subsets of images is implemented using the ‘flyweight’ pattern (Gamma *et al.*, 1994 ▶), whereby multiple subsets accessed through the lightweight high-level interface retain a reference to a single reader that performs the reading and interpretation. This has the advantage of reducing memory usage when accessing multiple subsets of images in parallel.

### 2.3. Image metadata storage   

A module is provided to enable straightforward storage of modified image set metadata. An image set may then be created from the file representation, allowing the refined experimental geometry to be saved for later use. The data are saved using the JavaScript object notation (JSON) format (Crockford, 2006 ▶); this format was chosen as it is human readable, is an open standard and is natively supported in many programming languages. In particular, the Python standard library contains a module for reading and writing arbitrary Python structures to JSON format, making it convenient for use within the *dxtbx*.

### 2.4. The Format plugin system   

The *dxtbx* provides a plugin mechanism to handle input from multiple file formats with alternative descriptions of the experimental geometry. Each 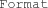 class is used to interpret a particular image file and metadata format, and a collection of 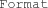 classes for common detectors and data representations are included as part of the *dxtbx*. Users may add their own to handle bespoke image formats or local variants (see Appendices *A* [App appa] and *B* [App appb]). A registry maintains a tree structure of these 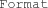 classes, such that the most specialized formats lie furthest from the root. Further details on the plugin mechanism can be found in Appendix *D* [App appd]. This model for handling different data representations has two advantages: no external site file is required for operation; furthermore, complex corrections (*e.g.* tile position corrections for a Pilatus detector) can be encoded in a self-contained way.

Extensibility of beamline descriptions was a key requirement in the development of the *dxtbx*: in particular, the ability for a beamline scientist to write a 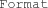 module (possibly extending a more general example for the detector) that describes how the values in the image header are to be used. Custom 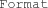 modules can be placed in a designated directory and are then automatically registered for use within the *dxtbx* on application startup. The ability to extend the library is primarily useful either where an unusual piece of experimental hardware is present or if the beamline has some idiosyncrasies, for example a left-handed rotation axis. Two examples will be used to demonstrate the ease with which the library may be extended.

#### 2.4.1. Example 1: reversed rotation axis   

The MX1 beamline at the Australian Synchrotron has a goniometer with left-handed rotation – the reverse of the conventional right-handed axis – but is an otherwise conventional beamline including an ADSC Quantum 210r detector, simply meaning that the direction of the rotation axis needs to be reversed. Within the *dxtbx* this is achieved by creating the Python file in Appendix *A* [App appa], which takes as a basis the standard 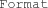 class for the ADSC detector and replaces the definition of the rotation axis, after ensuring (based on the detector serial number) that this is appropriate for these data.

#### 2.4.2. Example 2: ADSC Q315 on a 2θ arm   

The majority of ADSC CCD detectors are mounted on simple translation stages: given the size and weight of these devices there are rarely circumstances where more complex axes are needed. However, at ALS beamline 8.3.1 the Quantum 315 detector is mounted on a 2θ arm, which must be taken into consideration when processing the data. Here the beam centre recorded in the image header corresponds to the 2θ offset value rather than the position where the 2θ angle is 0° (James Holton, private communication). The 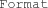 class to support this, included in Appendix *B* [App appb], replaces the detector definition to account for the shift in the detector origin and the changes in the vectors defining the detector plane resulting from the offset in 2θ. It is important to note that the changes are limited to the detector geometry, simplifying implementation for a beamline scientist, and will only affect detectors with a particular serial number (shown in the source code).

## 3. Applications   

The *dxtbx* aims to be generally applicable to the reading of image data and metadata for programs processing X-ray diffraction data. It has already found utility within established projects, such as the *cctbx* image viewer and *xia2*, as well as forming a key component in new projects, such as the *Diffraction Integration for Advanced Light Sources* (*DIALS*) project (Waterman *et al.*, 2013 ▶). Some examples showing simple usage of the *dxtbx* can be seen in Appendix *C* [App appc].

### 3.1. The *cctbx* image viewer   

The *cctbx* image viewer was designed to display diffraction images from a variety of diverse sources (Sauter *et al.*, 2013 ▶). It has been updated to utilize the *dxtbx*, showcasing the power of the 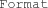, 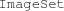 and 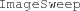 classes. When run from the command line, the viewer uses the *dxtbx* 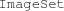 factory to create either a set or a sweep. It loads the first image in the set, displays it, and provides easy access to the rest of the files in the set by retaining a reference to the 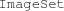 or 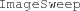 object. The *dxtbx* has allowed the application to quickly add support for several new file formats, most importantly newly defined HDF5 files.

HDF5 (The HDF Group, 2010 ▶) is a file container format currently being utilized in the context of large data sets such as those from XFEL beamlines or finely sliced synchrotron experiments. New generation detectors are currently supporting frame rates of the order of 120 Hz, and detectors on the horizon will be supporting frame rates of 1000 Hz or more. Depositing these data sets on the file system using a single file per image is not practical, making container technologies like HDF5 preferable. The *dxtbx* provides a plugin interface that allows the wrapping of an HDF5 data set in a 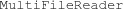 class, providing easy access to its contained images and metadata. As HDF5 formats evolve, new *dxtbx* plugins can be written or adapted to support their metadata formats (Brewster *et al.*, 2014 ▶). The plugins will seamlessly tie the new format to existing systems, allowing image display and processing.

### 3.2. *xia2*   

As mentioned in the *Introduction* [Sec sec1], *xia2* initially used the DiffractionImage library from the *CCP4* suite to read the headers from X-ray diffraction images. While this was effective for the initial range of experimental setups supported by the program, it increasingly became a limitation as more complex experimental geometries were supported, for example the use of κ goniometers and 2θ detector arms.

Initially this was addressed by providing alternative methods to read specific image types, which were tested in sequence after the DiffractionImage-based methods had failed; however, this approach quickly led to very complex code and scaled very poorly. Since the development and incorporation of the *dxtbx* into *xia2*, however, it has become much more straightforward to support analysis of arbitrary experimental geometries, allowing *xia2* to be used for the analysis of, for example, small molecule data (where more complex geometries are common) in addition to the macromolecular crystallography experiments it was designed for. In the future it is envisaged that this trend will continue, and that scientists developing new beamlines for crystallographic diffraction experiments will be able to add specific support for their beamlines themselves.

### 3.3. The *DIALS* framework   

The *DIALS* project aims to deliver an extensible framework and software package for the processing of diffraction data. It is intended for users of advanced light sources worldwide and, as such, is required to access image data and experimental geometries from a variety of data sources. To simplify the implementation and maintain generality, the experimental geometry and image data must be exposed in a uniform manner, independent of the underlying data representation. In the context of *DIALS*, the *dxtbx* provides a solution to these challenges.

## 4. Discussion   

The principle behind the *dxtbx* is to separate the interpretation of X-ray diffraction data from its analysis. Details of the experimental setup are encapsulated and exposed using a common interface and reference frame for all data types, ensuring that the client analysis code need not be aware of any file format specifics. The models produced by the *dxtbx* describe the key experimental components and may be used directly, with no further transformation. The *dxtbx* is also extensible in that a new experimental setup may be supported by the addition of a single Python file that describes the local environment: once this has taken place no changes should be needed within the *dxtbx* or the analysis code for the data to be correctly interpreted. Together these allow the developers of analysis code to focus on improving algorithms rather than the support of numerous detector data formats. Finally, the use of a completely general vectorial description of the experimental geometry allows for the propagation of detailed calibration information into the analysis code and may also encourage analysis software to support a similarly general approach to the processing of X-ray diffraction data.

## Footnotes

1 Currently the *dxtbx* supports only detectors made of a collection of flat rectangular sensors; support for truly curved instruments could, however, be added when the need arises.

## Appendix A Implementation: reversed rotation axis

Full implementation of a *dxtbx*  object, customized for Australian Synchrotron beamline MX1 with a reversed rotation axis.

## Appendix B Implementation: ADSC Q315 on a 2θ arm

Full implementation of a *dxtbx*  object for ALS beamline 8.3.1, where the ADSC Quantum 315 detector is mounted on a 2θ arm.

## Appendix C Simple examples

We briefly show some simple examples using the *dxtbx*. Full source code for these examples can also be found within the *dxtbx* source distribution under ./dxtbx/examples/.

### c1. Detector corner resolutions

A straightforward example that demonstrates the usefulness of the *dxtbx* is to compute the corner resolutions for a detector in an arbitrary position. Simply put, this example computes the 2θ angles between the beam and the position of each corner of the detector and converts them to the corresponding *d* spacing.

### c2. ImageSet interface

The code fragment below shows how the high-level  interface operates in a simple example. The  object, instantiated through a factory function, provides access to the experimental models and subsets of the , and allows simple indexing of images from the set or subset. Finally, an image volume can be easily extracted from the sequence of images contained in the .

### c3. Storage

The *dxtbx* provides a module to write a sweep or imageset to a JSON file. Either reading or writing an imageset from/to a file can be achieved in a single line of code. It should be noted that these functions do not read and write the image data directly; they read and write a representation that the *dxtbx* can use to understand the image data and metadata. The JSON format also loads any models that have been overridden in the imageset, allowing refined geometry for a sweep to be saved and loaded as necessary.

### c4. Detector-ray intersection

The detector model provides methods to calculate the point at which a ray intersects with the virtual detector plane described by its internal geometric representation as shown in the code fragment below. This point is returned in millimetres from the zeroth pixel in both the fast and slow direction. The detector model also provides a millimetre-to-pixel (and pixel-to-millimetre) function, allowing arbitrarily complex mappings.

## Appendix D Plugin mechanism

Given an image file, the registry calls the  function of the classes directly derived from the base class, . If a format claims to understand the image, the image is passed to the  function of its subclasses. The procedure continues recursively until the most deeply nested compatible format is returned. If at any level of the hierarchy more than one subclass understands an image, an exception is raised, thereby ensuring a unique result.

The registry tree is established automatically using a Python metaclass, which allows control over class creation. When a  class is first discovered, the metaclass recursively ensures that it is registered with the list of direct descendants of its base class. Because the metaclass is tied to the  base class it is implemented only once, and all of its subclasses will benefit from the auto-registration it provides.
